# Cellular plasticity and heterogeneity underlying therapy resistance in 3D cervical cancer models

**DOI:** 10.1186/s41065-026-00668-9

**Published:** 2026-04-14

**Authors:** Mercedes Olvera-Valencia, Adriana Grijalva-Pérez, Oliver Millan-Catalan, Martha Estela Albino-Sánchez, Manuel Humberto Cháirez-Ramírez, Rocío Méndez-Martínez, Eduardo López-Urrutia, Carlos Pérez-Plasencia, Alejandro García-Carrancá

**Affiliations:** 1 Subdirección de Investigación Básica, Laboratorio de Virus y Cáncer, Instituto Nacional de Cancerología, Mexico City, 14080 Mexico; 2 Programa Institucional de Biomedicina Molecular, Escuela Nacional de Medicina y Homeopatía del Instituto Politécnico Nacional, CDMX, Mexico; 3 Laboratorio de Virus & Cáncer, Unidad de Investigación Biomédica en Cáncer, Instituto de Investigaciones Biomédicas, Universidad Nacional Autónoma de México & Instituto Nacional de Cancerología, Mexico City, Mexico; 4 Posgrado en Ciencias Biológicas, Universidad Nacional Autónoma de México, Unidad de Posgrado, Edificio D, 1° Piso, Circuito de Posgrados, Ciudad Universitaria, Coyoacán, CDMX, C.P. 04510 México; 5 Subdirección de Investigación Básica, Unidad de Investigación Biomédica en Cáncer, Instituto Nacional de Cancerología, Mexico City, 14080 Mexico; 6 Departamento de Genetica y Biologia Molecular, Centro de Investigacion y Estudios Avanzados del Instituto Politécnico Nacional (Cinvestav-IPN), , Mexico City, 07360 Mexico; 7 Laboratorio Nacional de Apoyo a la Evaluación de Productos Bióticos (LaNAEPBi), Unidad de Servicio, Tecnológico Nacional de México/I.T. de Durango (TecNM/ITD), Blvd. Felipe Pescador 1830 Ote, Durango, Dgo C.P. 34080 México; 8 Laboratorio de Genómica Funcional, Unidad de Biomedicina, Facultad de Estudios Superiores Iztacala, Universidad Nacional Autónoma de México, Tlalnepantla, 54090 Mexico

**Keywords:** 3D cultures, Cervical cancer, Drug testing

## Abstract

Despite the success of prophylactic vaccination and screening programs, cervical cancer remains a major cause of cancer-related morbidity and mortality worldwide, particularly in low- and middle-income countries. In cervical cancer, therapeutic resistance cannot be explained by viral status alone and instead reflects broader tissue level and microenvironmental determinants. Increasing clinical and experimental evidence indicates that these processes are driven by dynamic cellular plasticity and microenvironmental adaptation rather than fixed genetic alterations alone. Three-dimensional (3D) model systems, including multicellular spheroids and patient-derived organoids, have transformed the study of cervical cancer by preserving tissue architecture, epithelial hierarchy, and spatial gradients of oxygen, nutrients, and signaling cues that are lost in conventional two-dimensional cultures. These models reveal how hypoxia, metabolic reprogramming, extracellular matrix interactions, and immune modulation converge to promote reversible, stress-tolerant cell states with stem-like features. In particular, 3D systems uncover hypoxia-associated redox adaptation, enhanced DNA damage repair capacity, and sustained viral oncogene expression within spatially defined niches that exhibit reduced sensitivity to cisplatin and radiotherapy. Here, we synthesize current advances in 3D cervical cancer modeling to illustrate how these platforms enable direct observation of resistance mechanisms that remain inaccessible in 2D systems. We discuss how organoids and advanced 3D culture systems provide mechanistic insight into plasticity programs operating in HPV-associated cervical cancer, tumor microenvironment remodeling, and the emergence of therapy-resistant states, while offering improved translational relevance for drug testing. Collectively, this review positions 3D cell culture models as a key tool for dissecting the dynamic biology of cervical cancer resistance and for guiding the rational design of therapeutic strategies aimed at preventing relapse.

## Why cervical cancer requires advanced 3D models

Despite being one of the most preventable malignancies through effective vaccination and screening programs, cervical cancer remains a leading cause of cancer-related morbidity and mortality worldwide, disproportionately affecting women in low- and middle-income countries [1]. While persistent infection with high-risk human papillomavirus (HPV) is an initiating event in cervical carcinogenesis, it is insufficient to fully explain tumor progression, therapeutic resistance, and disease recurrence, which emerge from complex interactions between viral oncogene activity, host cell plasticity, and the tumor microenvironment [2–4].Clinical data from cervical cancer consistently show that tumors with similar histology, HPV genotype, and clinical stage can display markedly different responses to chemoradiotherapy, underscoring the contribution of tumor-intrinsic heterogeneity and microenvironmental regulation [5–8].

**Fig. 1:**
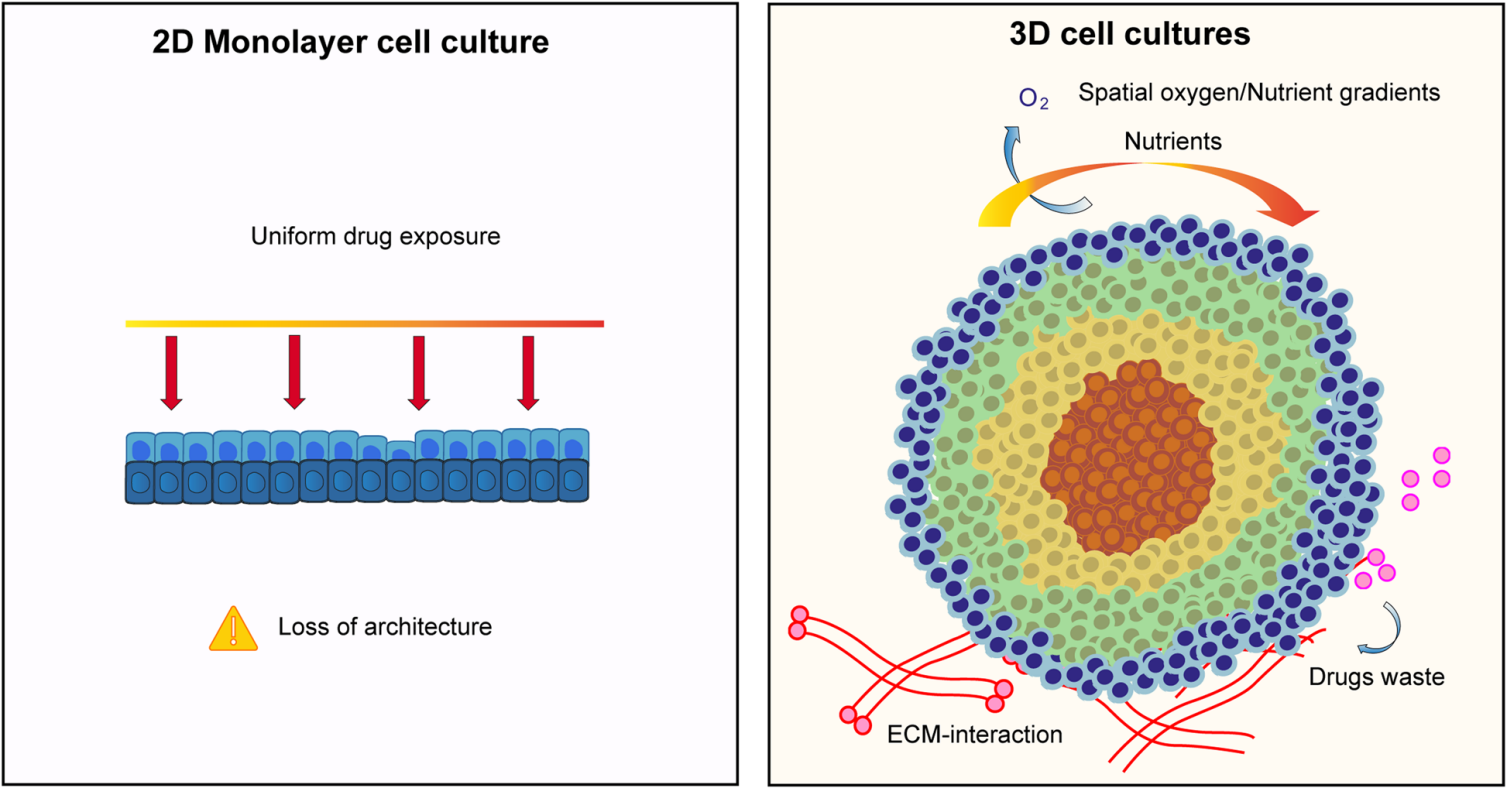
Comparison of 2D monolayer cultures versus 3D cell cultures in cervical cancer. **A** 2D Monolayer cell culture, characterized by uniform drug exposure and lack of structural complexity. Limitations include loss of tissue architecture, overestimation of drug efficacy, and an unstable HPV genome. **B** 3D cell culture. Recapitulates the physiological tumor microenvironment through spatial gradients of oxygen and nutrients. The model features extracellular matrix (ECM) transport barriers and regions of drug-tolerant plasticity and quiescence. Advantages include a physiological architecture, hypoxia core (red cells)

Standard experimental models have struggled to recapitulate these features. Two-dimensional (2D) monolayer cultures, which remain widely used in cervical cancer research, impose artificial growth conditions that rapidly erase epithelial identity, disrupt HPV gene regulation, and flatten cellular hierarchies [9, 10]. In these systems, cervical cancer cells are uniformly exposed to oxygen, nutrients, and drugs, leading to proliferation and an overestimation of therapeutic efficacy. Importantly, key resistance-associated phenotypes observed in cervical cancer—such as hypoxia tolerance, quiescence, altered DNA damage response, and enrichment of stem-like cell states—are either absent or severely attenuated in 2D cultures (Fig. 1A) [11–14].

Evidence from patient samples and clinical cohorts highlights that cervical tumors are spatially heterogeneous, with hypoxic niches enriched at the tumor core and invasive front, where cells display increased expression of stemness-associated markers, enhanced DNA repair capacity, and resistance to radiotherapy [15, 16]. These features are particularly relevant given that concurrent cisplatin-based chemoradiation remains the standard of care for locally advanced cervical cancer, and resistance to this regimen is a major determinant of poor outcome [5, 17–19]. The inability of 2D models to reproduce oxygen gradients and microenvironmental stress responses therefore represents a critical barrier to mechanistic understanding and therapeutic innovation [20–23]. Animal models provide a three-dimensional context but introduce additional limitations [24]. HPV-driven murine models often fail to fully recapitulate human cervical epithelial differentiation programs, transformation zone biology, and immune interactions [25]. Moreover, interspecies differences in stromal composition, immune surveillance, and viral persistence complicate the interpretation and translational relevance of *in vivo*findings [26, 27]. These limitations are particularly problematic when studying patient-specific variability and testing therapeutic strategies targeting dynamic cellular states rather than static genetic alterations [28, 29]. Three-dimensional (3D) culture systems directly address these gaps by providing a controllable yet physiologically relevant framework that preserves tissue architecture, spatial gradients, and cell–cell interactions characteristic of cervical tumors [11, 15, 30–34]. Spheroids and organoids derived from cervical cancer samples recapitulate hypoxic cores, nutrient gradients, and heterogeneous proliferative states that mirror those observed in patient tumors [11, 13, 35]. Crucially, these models maintain HPV genomes and more faithfully reproduce transcriptional programs, enabling investigation of viral/host interactions under conditions that resemble the *in vivo*niche. From a translational perspective, 3D models have demonstrated superior predictive power for therapeutic response in cervical cancer [36, 37]. Drug sensitivities observed in spheroids and patient-derived organoids correlate more closely with clinical outcomes than those measured in 2D cultures, particularly for agents whose efficacy is influenced by penetration, microenvironmental stress, or cell-cycle state [27, 36]. These systems also reveal that resistance is not solely a property of rare cancer stem cells but emerges from dynamic transitions between proliferative and stem-like states driven by microenvironmental cues—an insight that has profound implications for treatment design [38–40]. Taken together, accumulating clinical and experimental evidence indicates that the central challenges in cervical cancer—heterogeneity, plasticity, and resistance—are inherently three-dimensional problems [37]. Advanced 3D models therefore represent not merely an incremental improvement over conventional systems, but a necessary conceptual and experimental shift for accurately modeling cervical cancer biology and for developing therapies capable of overcoming resistance and reducing recurrence (Fig. 1B).

## Biological complexity of cervical cancer that 2D models fail to capture

### Transformation zone biology, reserve cells, and lineage plasticity

The cervix is characterized by a unique anatomical and functional region known as the squamocolumnar junction (SCJ) and the transformation zone (TZ), where squamous and columnar epithelia meet. This region is maintained by distinct populations of stem and reserve cells that support epithelial renewal and differentiation. Importantly, the TZ constitutes the predominant site where cervical neoplastic lesions arise, linking developmental lineage hierarchies and epithelial plasticity to cervical carcinogenesis [41].

**Fig. 2:**
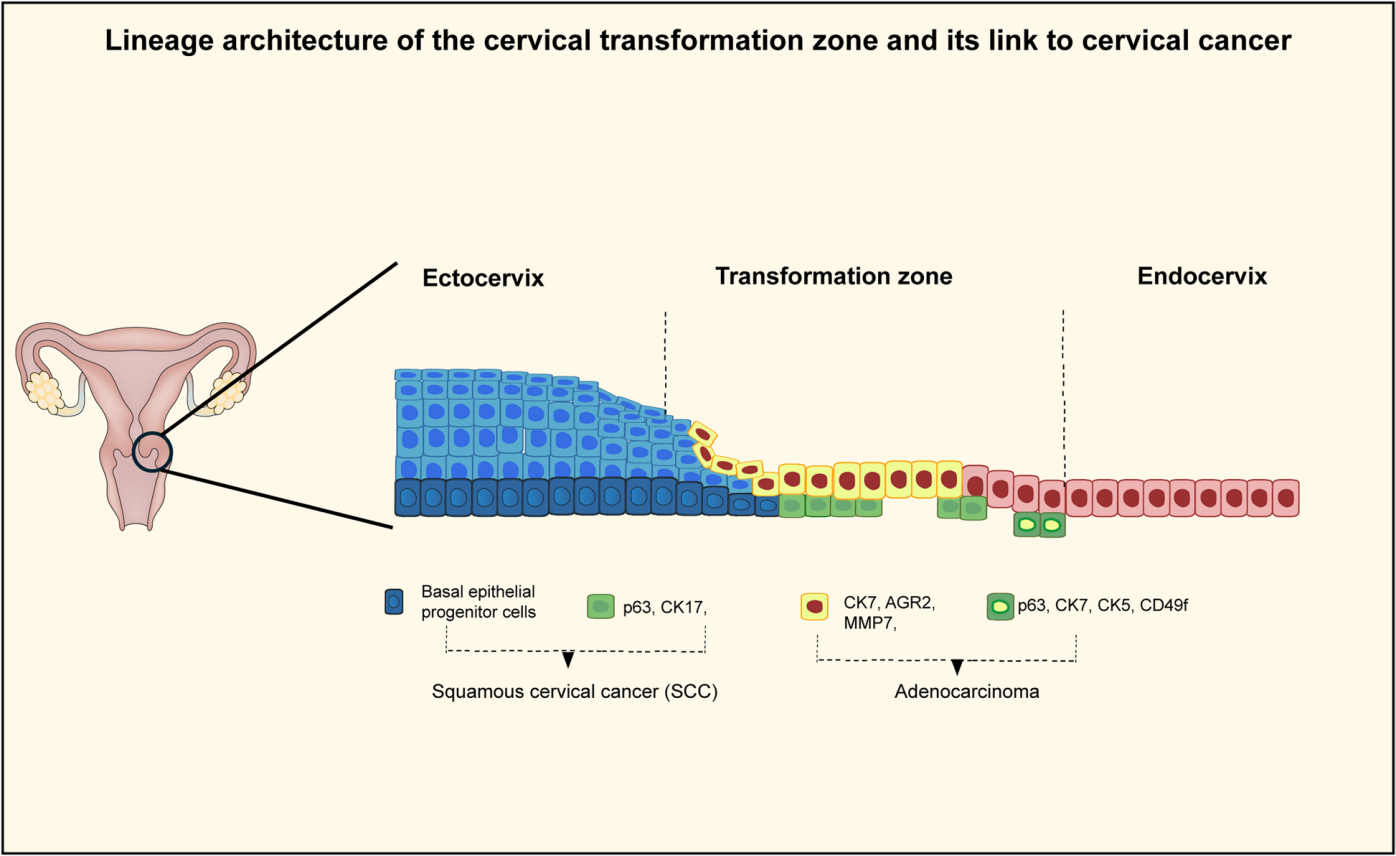
Lineage organization and plasticity at the cervical transformation zone. Schematic representation of the ectocervix, transformation zone (TZ), and endocervix, highlighting epithelial organization and lineage-associated marker expression. The ectocervix is composed of stratified squamous epithelium enriched for basal cells expressing p63 and CK17, whereas the endocervix consists of columnar epithelium characterized by CK7, AGR2, and MMP7 expression. The TZ represents a dynamic interface where transitional and metaplastic epithelial states coexist, including basal and progenitor-like populations expressing overlapping markers (e.g., p63, CK7, CK5, CD49f), reflecting lineage plasticity rather than fixed cell identities. These epithelial hierarchies are differentially associated with cervical cancer subtypes, with squamous cell carcinoma (SCC) predominantly linked to basal squamous lineages and adenocarcinoma associated with glandular and SCJ-related lineages. The schematic emphasizes cellular plasticity and spatial context without implying a single, deterministic cell of origin

Studies suggest that a discrete population of SCJ cells serves as the cell of origin for HPV-associated cervical cancer, based on persistent expression of lineage-specific markers such as KRT7, AGR2, CD63, MMP7, and GDA in invasive tumors [26]. However, this model was derived from static tissue analyses and does not account for lineage plasticity or microenvironment-driven state transitions, which have since emerged as key determinants of cervical tumor initiation and progression [26]. Evidence supporting epithelial plasticity at the cervical transformation zone comes from reprogramming and 3D culture studies. Sato et al. demonstrated that cervical reserve cell–like properties can be regenerated from human induced pluripotent stem cells (iPSCs), generating induced reserve cell–like cells (iRCs). [42] These cells expressed canonical reserve cell markers (p63, CK5, CK8) as well as SCJ-associated markers (CK7, AGR2, CD63, MMP7, GDA), indicating that SCJ-like molecular signatures can be acquired through induced differentiation rather than reflecting a fixed lineage [42]. Notably, iRCs also exhibited immunomodulatory features, including secretion of inflammatory cytokines and expression of HLA-G, suggesting that reserve/SCJ-like states may actively contribute to immune tolerance and tumor-supportive microenvironments (Fig. 2). These findings imply that progenitor-like epithelial states in the cervix are not only plastic but may also play a functional role in shaping tumor–immune interactions [42]. Consistent with this concept, Ortiz-Sánchez et al. reported that cervical cancer cells cultured under 3D sphere-forming conditions acquire stem-like phenotypes characterized by expression of CD49f, p63, ALDH, OCT4, NANOG, and β-catenin. Importantly, markers such as CD49f and AII were associated with increased susceptibility to high-risk HPV infection, reinforcing the notion that stem-like and HPV-permissive states emerge dynamically in response to microenvironmental and architectural cues rather than being confined to a pre-existing cell population [43]. Transition zones represent well-recognized hotspots for cancer emergence and are frequently preceded by metaplasia, a process in which one epithelial lineage replaces another [41]. Despite their clinical relevance, the mechanisms that maintain epithelial spatial organization and govern niche remodeling during metaplasia have remained incompletely understood [37, 41]. Using human cervical tissue and organoid-based approaches, Chumduri et al. demonstrated that ectocervical and endocervical epithelia derive from distinct, cervix-resident lineage-specific stem cell populations regulated by opposing stromal WNT signaling gradients. [27] Importantly, they showed that remodeling of the endocervical stroma, including increased expression of the WNT inhibitor Dickkopf-2 (DKK2), promotes the expansion of ectocervical stem cells, providing a mechanistic basis for epithelial replacement at the transformation zone. Although the cellular composition of the endocervix closely resembles that of the transformation zone, single-cell analyses revealed that epithelial, stromal, immune, and smooth muscle subpopulations within the ectocervix display distinct transcriptional programs. These spatially defined differences become progressively accentuated during disease progression, as cervical intraepithelial neoplasia (CIN I) evolves toward invasive cervical cancer. This transition is accompanied by widespread transcriptional reprogramming and microenvironmental remodeling, including the enrichment of stem-like phenotypes. Consistent with this, increasing expression of pluripotency-associated transcription factors such as NANOG, SOX2, and KLF4 has been observed alongside elevated levels of cervical cancer stem cell–associated markers, including CD133, CD44, ALDH1, CK17, p63, CK5/8, MSI1, CD49f, ABCG2, BMI1 and PIWIL2 [43]. While many of these markers are shared across cancer stem cell populations in different tumor types, their coordinated emergence in cervical cancer reflects activation of conserved regenerative programs rather than expansion of a fixed cell population [36]. The interaction between these cellular compartments establishes a hierarchical but highly plastic organisation in which tumour growth, persistence and recurrence are maintained by cells capable of reversibly entering proliferative and regenerative states. Rather than being sustained by a single cancer stem cell population, cervical cancer persistence emerges from a dynamic network comprising normal progenitors, SCJ-associated epithelial states, and stem-like tumor cells that collectively preserve tumor integrity and enable survival under therapeutic pressure [27, 36, 44, 45]. Understanding how these stem-like states contribute to therapy resistance is therefore essential for the development of more effective treatment strategies.

Schematic representation of the ectocervix, transformation zone (TZ), and endocervix, highlighting epithelial organization and lineage-associated marker expression. The ectocervix is composed of stratified squamous epithelium enriched for basal cells expressing p63 and CK17, whereas the endocervix consists of columnar epithelium characterized by CK7, AGR2, and MMP7 expression. The TZ represents a dynamic interface where transitional and metaplastic epithelial states coexist, including basal and progenitor-like populations expressing overlapping markers (e.g., p63, CK7, CK5, CD49f), reflecting lineage plasticity rather than fixed cell identities. These epithelial hierarchies are differentially associated with cervical cancer subtypes, with squamous cell carcinoma (SCC) predominantly linked to basal squamous lineages and adenocarcinoma associated with glandular and SCJ-related lineages. The schematic emphasizes cellular plasticity and spatial context without implying a single, deterministic cell of origin.

### Drivers of therapy resistance in cervical cancer: dynamic stem-like states

Regardless of the cellular origins and intratumoral heterogeneity of cervical cancer, a critical challenge lies in understanding how stem-like and progenitor-like cell states translate their unique biological properties into concrete mechanisms of therapy resistance. Dynamic populations actively participate in adaptive programmes that enable survival under therapeutic pressure, including enhanced DNA repair, redox homeostasis, apoptosis evasion, drug efflux, and entry into resting states. Importantly, many of these resistance mechanisms become fully evident only when cervical cancer cells are studied in three-dimensional (3D) contexts that preserve tissue architecture, spatial gradients, and microenvironmental stress.

### Intrinsic cellular mechanisms

One of the most consistently reported intrinsic resistance mechanisms associated with stem-like states in cervical cancer is enhanced activation of DNA damage response (DDR) pathways [46]. Cervical cancer cells enriched for stemness markers display increased proficiency in repairing therapy-induced DNA lesions, particularly double-strand breaks generated by radiotherapy and platinum-based chemotherapy. This phenotype is supported by upregulation of key homologous recombination (HR) and non-homologous end joining (NHEJ) components, including RAD51 and BRCA1, linking stem-like programs to efficient DNA repair and reduced treatment-induced cell death [47]. Functional studies demonstrate that CSC-enriched populations—identified by markers such as ALDH1 and CD133—exhibit accelerated DNA repair kinetics and diminished apoptosis following genotoxic stress. Notably, inhibition of DDR effectors, including RAD51, sensitizes these stem-like populations to cisplatin and radiotherapy, underscoring DDR dependency as a critical vulnerability [47]. Importantly, these resistance phenotypes are more robustly revealed in 3D spheroid and organoid models, where hypoxia, cell-cycle heterogeneity, and restricted drug penetration more accurately recapitulate clinical treatment conditions than conventional 2D cultures [10]. Redox adaptation represents a second major axis of intrinsic resistance. While standard therapies involve inducing reactive oxygen species (ROS) to cause DNA damage and apoptosis, cervical cancer stem cells maintain lower basal ROS levels compared to more differentiated tumor cells. This redox balance is achieved through upregulation of antioxidant systems, including glutathione (GSH), superoxide dismutase (SOD), catalase, and peroxiredoxins, which collectively buffer oxidative stress and preserve stem-associated phenotypes [48]. In cervical cancer, ALDH1-positive populations show elevated expression of antioxidant genes, correlating with resistance to both cisplatin and radiotherapy. Hypoxic tumor niches further reinforce this phenotype through stabilization of HIF-1α, which promotes metabolic reprogramming and enhances antioxidant capacity, facilitating the persistence of quiescent, therapy-tolerant cell states [49]. Three-dimensional culture systems reveal that these redox-adapted phenotypes are spatially restricted, with cells residing in hypoxic spheroid or organoid cores exhibiting heightened antioxidant defenses and survival advantages that are largely absent in 2D monolayers [50]. Evasion of apoptosis constitutes an additional hallmark of stem-like states in cervical cancer. SOX2-positive populations, in particular, display marked resistance to apoptosis and are associated with poorer clinical outcomes, directly linking stemness-associated transcriptional programs to therapeutic failure [51]. Rather than reflecting a static cancer stem cell pool, these apoptosis-resistant phenotypes emerge dynamically in response to microenvironmental stress and treatment exposure—a process that is faithfully recapitulated only in 3D systems that preserve cell–cell interactions and niche-derived survival cues [52, 53]. Together, these observations underscore that therapy resistance in cervical cancer is an emergent, spatially organized phenomenon driven by dynamic cellular states. The dependence of these mechanisms on architectural context highlights the limitations of 2D models and establishes advanced 3D systems as essential tools for dissecting resistance biology and identifying effective therapeutic vulnerabilities.

### Tumor microenvironment and HPV-driven remodeling

Persistent infection with high-risk human papillomavirus (HPV) is not only the initiating event in cervical carcinogenesis but also a sustained driver of cellular plasticity, microenvironmental remodeling, and therapy resistance. Continuous expression of the viral oncoproteins E6 and E7 enforces cell-cycle deregulation through degradation of p53 and functional inactivation of RB, thereby uncoupling proliferation from differentiation and predisposing infected cells to genomic instability [46, 54]. Beyond these canonical effects, HPV actively reprograms epithelial cell states. E6 and E7 promote the acquisition of stem-like phenotypes by activating pluripotency-associated transcriptional programs, including SOX2, OCT4, and NANOG, and by reinforcing epithelial–mesenchymal transition (EMT) [26]. These programs increase cellular plasticity, allowing infected cells to reversibly transition between proliferative, quiescent, and invasive states in response to microenvironmental stress and therapeutic pressure [55]. HPV also reshapes the tumor microenvironment through multiple immunomodulatory mechanisms. Viral oncoproteins impair antigen presentation by downregulating MHC class I molecules [56, 57], suppress interferon signaling, and promote the secretion of immunosuppressive cytokines [58, 59]. These effects favor the recruitment and polarization of tumor-associated macrophages toward an M2 phenotype, expansion of myeloid-derived suppressor cells, and accumulation of regulatory T cells, collectively establishing an immune-tolerant niche that supports the survival of infected, therapy-resistant cell populations [60, 61]. Metabolic and hypoxic adaptations further amplify HPV-driven resistance. Stabilization of HIF-1α in HPV-positive cells enhances glycolytic metabolism, lactate production, and antioxidant capacity, reinforcing redox balance and promoting resistance to radiotherapy and chemotherapy. These adaptations are spatially restricted and particularly evident in hypoxic tumor regions, where HPV-infected cells exhibit reduced proliferation, enhanced DNA repair capacity, and diminished sensitivity to cytotoxic agents [20, 62]. Importantly, many of these HPV-driven effects are underestimated or entirely missed in two-dimensional culture systems [10], where viral gene expression, immune interactions, and spatial gradients are poorly maintained. In contrast, three-dimensional models preserve HPV episomes, sustain viral oncogene expression, and recapitulate the architectural and metabolic constraints of the cervical tumor niche [25, 37]. As a result, 3D systems reveal HPV-driven remodeling as a dynamic and spatially organized process that directly contributes to the emergence and maintenance of therapy-resistant cell states [63].

Collectively, these observations underscore that therapy resistance in cervical cancer is not solely dictated by fixed genetic alterations but emerges from dynamic, spatially regulated interactions between HPV-driven epithelial programs, stem-like cell states, and the tumor microenvironment. Such complexity—characterized by cellular plasticity, hypoxic and metabolic gradients, immune modulation, and lineage-specific responses—cannot be captured by conventional two-dimensional culture systems or reductionist experimental approaches. Consequently, there is a critical need for advanced experimental platforms capable of preserving epithelial hierarchy, viral persistence, and microenvironmental constraints. In this context, three-dimensional (3D) model systems have emerged as indispensable tools for studying cervical cancer initiation, progression, and therapy resistance.

## Overview of advanced 3D model platforms in cervical cancer

The cellular origin of cervical cancer remains incompletely defined, although the cervical transformation zone (TZ) represents a well-recognized anatomical and biological hotspot for HPV-associated neoplastic transformation [53, 64, 65].Persistent infection with oncogenic HPV requires long-lived epithelial cell populations capable of maintaining viral genomes over extended periods, suggesting that progenitor or stem-like cell states may contribute to tumor initiation without being uniquely restricted to a single, well-defined cell population [4, 55, 63]. Importantly, not all tumor cells contribute equally to disease progression, and only specific subpopulations sustain long-term growth and tumor propagation [4, 66–71]. Consequently, experimental models of cervical cancer must preserve cellular plasticity, lineage diversity, and intratumoral heterogeneity rather than assuming a fixed cell of origin [52, 72]. Traditional preclinical approaches, including xenografts derived from established cell lines or primary tumors implanted into immunodeficient mice, provide a valuable three-dimensional context that incorporates vascularization and stromal interactions [11].These models have been instrumental in uncovering tumor–stroma dynamics underlying cervical carcinogenesis. However, they remain costly, time-consuming, and limited in their capacity to fully capture patient-specific heterogeneity [73]. Similarly, genetically engineered mouse models often fail to faithfully reproduce the spatial organization, lineage dynamics, and HPV-driven processes observed in human disease [74].

Three-dimensional (3D) *in vitro*models have emerged to address these limitations. Cervical cancer spheroids derived from established cell lines enrich for stemness-associated populations (e.g., ALDH1⁺, CD44⁺, SOX2⁺), exhibit increased resistance to cisplatin and radiotherapy, and survive under hypoxic stress, recapitulating core features of therapy-resistant subpopulations. Nevertheless, spheroids alone cannot fully represent the cellular diversity or architectural complexity of cervical tumors [75]. Organoids represent a more advanced and physiologically relevant platform. These cultures preserve the histopathological and molecular characteristics of the tumor of origin while maintaining subclonal diversity. Importantly, tumor-derived organoids can often be paired with organoids generated from matched normal cervical tissue, providing individualized controls for mechanistic and therapeutic studies [11, 36, 37, 66].

A major breakthrough occurred in 2019, when Maru et al. reported the first patient-derived cervical cancer organoids, demonstrating their utility for interrogating EMT-associated vulnerabilities. [35] Subsequently, Löhmussaar *et al.*established organoids from both ectocervical and endocervical tissues of healthy individuals and cervical cancer patients, revealing lineage-specific growth factor dependencies and divergent responses to WNT signaling [36]. Notably, R-spondin–mediated WNT activation inhibited ectocervical organoid expansion while being essential for endocervical organoid maintenance, mirroring stromal WNT regulation observed in vivo [36]. These findings were later integrated with *in vivo*data demonstrating that ectocervical and endocervical epithelia arise from lineage-restricted stem cell populations governed by opposing stromal WNT signals. High expression of the WNT inhibitor Dickkopf-2 (DKK2) in the endocervical stroma promotes differentiation of CK5⁺ reserve cells toward squamous epithelium in the transformation zone, a process central to squamous metaplasia and a recognized precursor state for HPV-driven neoplastic transformation [28]. Organoid based analyses have contributed to the ongoing debate regarding the cellular origin of cervical squamous cell carcinoma by enabling lineage informed comparisons under physiologically relevant conditions, comparative transcriptomic analyses revealed that ectocervical organoids and TCGA squamous cervical cancers display high KRT5 expression and low levels of squamocolumnar junction (SCJ) markers, whereas endocervical organoids and adenocarcinomas retain SCJ-associated signatures [27]. These observations argue against SCJ cells as the direct cells of origin for squamous carcinoma and instead support lineage continuity from ectocervical progenitors. Further advances include the generation of organoids from Pap brush samples, enabling the establishment of biobanks that retain causative HPV genomes and enabling patient-specific drug testing. More recently, the introduction of uterine cervix extracellular matrix (UCEM) hydrogels has improved transcriptional fidelity relative to Matrigel-based cultures and revealed enhanced chemoresistance, underscoring the importance of tissue-specific matrices for modeling therapeutic response [11]. Collectively, these developments position cervical organoids as biologically faithful models of cervical stem cell lineages and HPV-driven tumor evolution. By recapitulating lineage-specific signaling, spatial organization, and viral persistence, advanced 3D platforms provide a powerful framework for studying therapy resistance and for advancing precision oncology in cervical cancer. Remaining challenges include protocol standardization, long-term genomic stability, and incorporation of immune and stromal compartments—critical steps for translating organoid technology into robust preclinical and clinical applications.

## How 3D Models reveal dynamic resistance mechanisms

Three-dimensional (3D) model systems have enabled the direct observation of resistance mechanisms in cervical cancer that remain inaccessible in two-dimensional cultures [35, 36, 50]. By imposing spatial constraints, epithelial stratification, and microenvironmental gradients, 3D models reveal how therapy resistance emerges from dynamic interactions between HPV-driven programs, stem-like cell states, and local metabolic and hypoxic stress rather than from static genetic alterations alone [36, 37].

### Spatial Control of DNA Damage Responses in Hypoxic Niches

Hypoxia is a defining microenvironmental feature of cervical cancer and a well-established determinant of poor therapeutic response [20]. Clinical and experimental studies have consistently shown that oxygen deprivation within cervical tumors is spatially heterogeneous, generating distinct cellular niches characterized by reduced proliferation, metabolic rewiring, and enhanced survival capacity [20–22, 62]. These hypoxic regions stabilize hypoxia-inducible factors, particularly HIF-1α, which orchestrate transcriptional programs that promote glycolytic metabolism, angiogenic signaling, redox homeostasis, and resistance to both radiotherapy and chemotherapy [17, 76]. In cervical cancer, hypoxia has been directly associated with decreased sensitivity to radiotherapy, largely due to impaired reactive oxygen species (ROS) generation and attenuated fixation of radiation-induced DNA damage [20]. Hypoxic tumor regions also exhibit altered cell-cycle dynamics, with a shift toward quiescent or slowly cycling states that further reduce susceptibility to cytotoxic therapies [49]. Clinically, elevated HIF-1α expression and markers of tumor hypoxia correlate with poor local control, increased recurrence, and reduced overall survival, underscoring the central role of oxygen gradients in shaping treatment outcome [22, 77]. Beyond these canonical effects, hypoxia profoundly remodels DNA damage response pathways in cervical cancer [78]. Cells residing in hypoxic niches display enhanced activation of homologous recombination repair mechanisms, including increased expression and activity of RAD51 and BRCA1, which facilitate efficient repair of double-strand breaks induced by ionizing radiation or platinum-based chemotherapy [79, 80]. This hypoxia-driven enhancement of DNA repair capacity contributes to the persistence of therapy-resistant cell populations and supports tumor regrowth following treatment [81]. Importantly, these adaptations are spatially restricted and emerge specifically within poorly oxygenated tumor regions, highlighting hypoxia as a localized, rather than global, driver of resistance [10, 80–82]. In the context of HPV-associated cervical cancer, hypoxia intersects with viral oncogene–mediated programs in ways that further reinforce resistance [81]. HPV-positive cells maintain expression of the viral oncoproteins E6 and E7 under hypoxic conditions, enabling continued disruption of p53 and RB pathways even in quiescent or metabolically stressed states [83]. This sustained oncogene expression supports cell survival, limits apoptosis, and cooperates with hypoxia-induced DNA repair and antioxidant programs. As a result, hypoxic niches may serve as reservoirs of long-lived, HPV-infected cells that are particularly adept at surviving therapeutic insults and contributing to disease recurrence [84]. Crucially, many of these hypoxia-driven processes are underestimated or entirely absent in conventional two-dimensional culture systems. In 2D monolayers, uniform oxygenation prevents the formation of physiologically relevant oxygen gradients and precludes stabilization of HIF-1α, masking hypoxia-dependent metabolic and DNA repair programs. Consequently, 2D models fail to capture the spatial segregation of proliferative and resistant cell populations that is observed *in vivo* [85]. In contrast, three-dimensional spheroids and organoid models of cervical cancer intrinsically develop oxygen diffusion limitations that give rise to hypoxic cores surrounded by more oxygenated peripheral regions [85].These systems faithfully reproduce spatially resolved hypoxia, enabling direct investigation of how oxygen gradients shape cellular state, DNA repair capacity, and therapeutic response. Within 3D cervical cancer models, cells localized to hypoxic regions exhibit reduced proliferation, enhanced activation of DNA damage response pathways, sustained HPV oncogene expression, and diminished sensitivity to cisplatin and radiotherapy—features that closely mirror clinical observations [84, 86–89].Together, these findings position hypoxia not merely as a passive consequence of tumor growth but as an active, spatially organized driver of therapy resistance in HPV-associated cervical cancer. Three-dimensional model systems provide a uniquely powerful platform to dissect the dynamic interplay between hypoxia, viral oncogene activity, and cellular plasticity, offering critical insights into resistance mechanisms that are inaccessible in two-dimensional cultures. Incorporating hypoxia-aware 3D models into preclinical research is therefore essential for the rational design of therapeutic strategies aimed at overcoming resistance and improving durable treatment responses in cervical cancer [84, 86–89].

### Metabolic reprogramming and redox adaptation revealed in 3D

Three-dimensional (3D) cervical cancer models have revealed metabolic and redox adaptations that are largely obscured in two-dimensional (2D) culture systems. Foundational work using multicellular tumor spheroids established that diffusion-limited transport of oxygen and nutrients in 3D architectures generates spatially organized metabolic heterogeneity, including hypoxic, glycolytic, and redox-adapted cell populations that closely resemble solid tumors *in vivo*—features inherently absent in 2D monolayers [86]. This conceptual framework provides a mechanistic basis for understanding why cervical cancer organoids stabilize stress-adaptive metabolic states relevant to therapeutic resistance. Patient-derived organoids (PDOs) from cervical tumors retain key transcriptional, epigenetic, and functional features of the tissue of origin, including signaling pathways that intersect with metabolic regulation and therapy response. Early work by Maru et al. (2019)demonstrated that cervical cancer organoids can be robustly established from patient samples while preserving histopathological characteristics and oncogenic pathway activity. Although the primary focus of this study was organoid derivation and drug sensitivity, differential responses to EMT- and pathway-targeted inhibitors were observed in 3D but not in matched 2D cultures, consistent with underlying metabolic plasticity and redox-buffering capacities enabled by 3D architecture [35]. Based on this, Lohmussaar et al. (2021) generated organoids from both healthy cervical epithelium and cervical carcinomas and revealed lineage-specific dependencies on WNT signaling. Importantly, WNT pathway activity is tightly coupled to cellular metabolism, antioxidant defenses, and stem-like behavior. By preserving epithelial hierarchy and spatial organization, these organoids exhibited heterogeneous cellular states that likely reflect gradients of nutrients and oxygen analogous to those described in classical spheroid systems. While metabolic fluxes were not directly quantified, the maintenance of WNT-dependent stem-like populations in 3D supports a metabolic configuration compatible with redox balance, quiescence, and long-term survival under stress [36]. More recently, Song et al. (2024) provided direct experimental evidence that extracellular matrix (ECM) composition critically shapes metabolic adaptation and drug resistance in cervical cancer organoids. Organoids cultured in uterine cervix–derived ECM hydrogels displayed transcriptional programs more closely aligned with patient tumors than Matrigel-grown counterparts, including enrichment of pathways associated with oxidative stress responses, metabolic regulation, and chemoresistance. Notably, these ECM-supported organoids exhibited increased carboplatin IC₅₀ values, indicating enhanced drug tolerance that correlated with preserved oncogene expression and tissue-specific metabolic constraints [11]. Collectively, these studies demonstrate that cervical cancer organoids do not merely recapitulate tumor morphology but uncover metabolically and redox-adapted states that are inaccessible in 2D systems. Consistent with principles established in classical tumor spheroid models, 3D architecture enables the stabilization of metabolic heterogeneity, antioxidant defenses, and stress-tolerant cell populations that directly modulate therapeutic sensitivity. These insights position 3D cell culture as indispensable tool for dissecting metabolic vulnerabilities and for the rational design of therapies aimed at overcoming redox-mediated resistance in cervical cancer [40, 86, 90, 91].

### Reversible drug-tolerant states and stem-like plasticity

Unlike conventional two-dimensional (2D) cultures, three-dimensional (3D) models reveal that therapy resistance in cervical cancer frequently arises from reversible, non-genetic drug-tolerant states rather than fixed genetic alterations. Within spheroids and organoids exposed to chemoradiotherapy, discrete subpopulations of cells adopt quiescent or slow-cycling phenotypes that are transiently refractory to treatment. These states are characterized by enrichment of stemness-associated transcriptional programs, including increased expression of SOX2, OCT4, and NANOG, together with activation of epithelial–mesenchymal transition (EMT)–related pathways, reflecting heightened cellular plasticity [35, 36, 86]. Importantly, these drug-tolerant states are functionally reversible. Upon treatment withdrawal, quiescent cells re-enter the proliferative pool, repopulate the 3D structure, and restore cellular heterogeneity, thereby recapitulating key features of tumor relapse observed clinically [11]. This dynamic behavior is difficult to capture in 2D monolayers, where forced proliferation, uniform nutrient availability, and the absence of spatial constraints suppress the emergence and maintenance of slow-cycling populations [40, 92].The plasticity observed in 3D models closely mirrors HPV-driven epithelial reprogramming in cervical cancer. Persistent expression of HPV oncoproteins sustains undifferentiated cell states and facilitates reversible transitions between proliferative, quiescent, and invasive phenotypes in response to environmental stress and therapeutic pressure [36]. By preserving tissue architecture, oxygen and nutrient gradients, and cell–cell interactions, 3D systems provide a physiologically relevant framework in which these HPV-associated adaptive programs can be stabilized and interrogated. Together, these findings position reversible drug-tolerant states as a central mechanism underlying therapeutic failure and disease recurrence in cervical cancer. Advanced 3D culture platforms therefore offer a critical experimental window into stem-like plasticity, enabling the identification of vulnerabilities that may be exploited to prevent tumor regrowth following initial treatment response.

### Limitations and unresolved questions in 3D cervical cancer models

Despite their conceptual and experimental advantages, current 3D cervical cancer models remain incomplete representations of tumor biology. Most organoid and spheroid systems lack functional vascularisation, immune cell dynamics, and widespread stromal remodelling, limiting their ability to model inflammation induced by treatment, immune clearance, and systemic therapeutic responses [75, 84, 93]. Furthermore, *in vitro* oxygen and nutrient gradients are driven by diffusion and do not fully reproduce the perfusion-dependent heterogeneity observed *in vivo* [85, 86]. Another important limitation concerns causality. While 3D models reveal strong associations between hypoxia, stem-like states, and therapy resistance, many studies remain correlative and do not yet establish direct causal hierarchies between microenvironmental stress, viral oncogene regulation, and resistance phenotypes [81, 82]. Similarly, long-term evolutionary dynamics such as clonal selection under therapy pressure are only partially captured in current culture systems. Finally, variability in extracellular matrix composition, culture protocols, and organoid derivation methods introduces experimental heterogeneity that complicates cross-study comparisons. These limitations highlight the need to integrate 3D models with complementary systems, including immune-competent platforms, organ-on-chip technologies, and *in vivo*validation frameworks [94, 95].

### Nanoparticles and therapeutic testing in 3D cervical cancer models

Nanoparticle-based therapeutic strategies should be interpreted within the broader framework of spatially organized resistance revealed by cervical cancer 3D models. Because drug penetration, cellular uptake, and therapeutic response are strongly modulated by hypoxia, extracellular matrix composition, and stem-like cell states [96]. Importantly, while several nanoparticle transport principles described below derive from studies in other solid tumor spheroid systems, emerging evidence from cervical cancer organoids indicates that metabolic adaptation, extracellular matrix composition, and CSC-enriched niches impose similar transport constraints. Thus, cervical-specific 3D models provide a necessary platform for translating nanoparticle design principles into disease-relevant therapeutic strategies [10, 80]. Consequently, therapeutic strategies aimed at eliminating or reprogramming CSCs have emerged as promising approaches to improve durable treatment responses. Nanoparticle-based delivery systems represent a particularly attractive platform for this purpose, enabling the targeted delivery of cytotoxic drugs, siRNA/miRNA, immunomodulators, or combination therapies designed to simultaneously address CSCs and non-stem tumor cells. Polymeric, lipid-based, and inorganic nanoparticles can be engineered to optimize solubility, circulation time, active targeting, and controlled or stimuli-responsive drug release [97, 98]. Recent preclinical studies have explored nanoparticles delivering siRNA/miRNA targeting stemness pathways, co-delivery of chemotherapeutics with CSC-directed agents such as salinomycin, and surface functionalization with ligands targeting CSC-associated receptors, including CD44 and integrins [97, 98]. However, a major translational barrier persists: nanoparticle formulations that demonstrate high efficacy in 2D monolayer cultures frequently fail to reproduce similar therapeutic benefit *in vivo*. Growing consensus attributes this discrepancy to the inability of 2D systems to capture the structural complexity, cellular heterogeneity, and microenvironmental features of tumors, particularly those associated with CSC niches, extracellular matrix (ECM) architecture, and hypoxia [99]. As nanoparticle transport, uptake, and pharmacodynamics are highly sensitive to these factors, reliance on 2D culture alone can lead to misleading conclusions regarding translational potential, where cervical-specific evidence is limited, mechanistic insights are extrapolated from structurally comparable solid tumor spheroid systems [100, 101].

### Physical transport of nanoparticles in 2D and 3D systems

In 2D monolayer cultures, nanoparticles encounter a relatively uniform aqueous environment and direct access to cell membranes, such that apparent delivery efficiency is dominated by simple diffusion and receptor-mediated internalization. In contrast, 3D spheroids and organoids recreate tissue-like architectures that impose steric hindrance, electrostatic interactions, and tortuous diffusion pathways. As a result, nanoparticle distribution in 3D models is highly heterogeneous and more predictive of intratumoral distribution observed in xenografts and patient tumors. Across multiple polymeric nanoparticle systems, several consistent transport principles have emerged. Particle size is a dominant determinant of penetration depth, with smaller nanoparticles (<100 nm) generally diffusing more effectively into spheroids than larger counterparts. Importantly, particle deformability can modulate this relationship, as soft or flexible polymeric micelles may penetrate more deeply than rigid nanoparticles of comparable hydrodynamic size. Surface chemistry also plays a critical role: strongly cationic particles tend to bind negatively charged ECM components and become sequestered at the spheroid periphery, whereas neutral or PEGylated surfaces reduce nonspecific ECM interactions and enhance penetration [102–104]. While interstitial convection influences nanoparticle transport *in vivo*, diffusion dominates in compact spheroids, making them particularly useful for isolating diffusion-limited transport phenomena. Tumor-on-chip platforms incorporating controlled flow further demonstrate that convection increases peripheral accumulation but rarely improves penetration into dense tumor cores, reinforcing the importance of ECM-mediated diffusion barriers [1]. These insights underscore the need for nanoparticle design strategies tailored specifically to 3D transport constraints, including size minimization, stealth coatings, tumor-penetrating peptides, and protease-responsive ECM-modulating approaches [105, 106].

### Cellular uptake and endocytic pathways in 3D CSC models

**Fig. 3:**
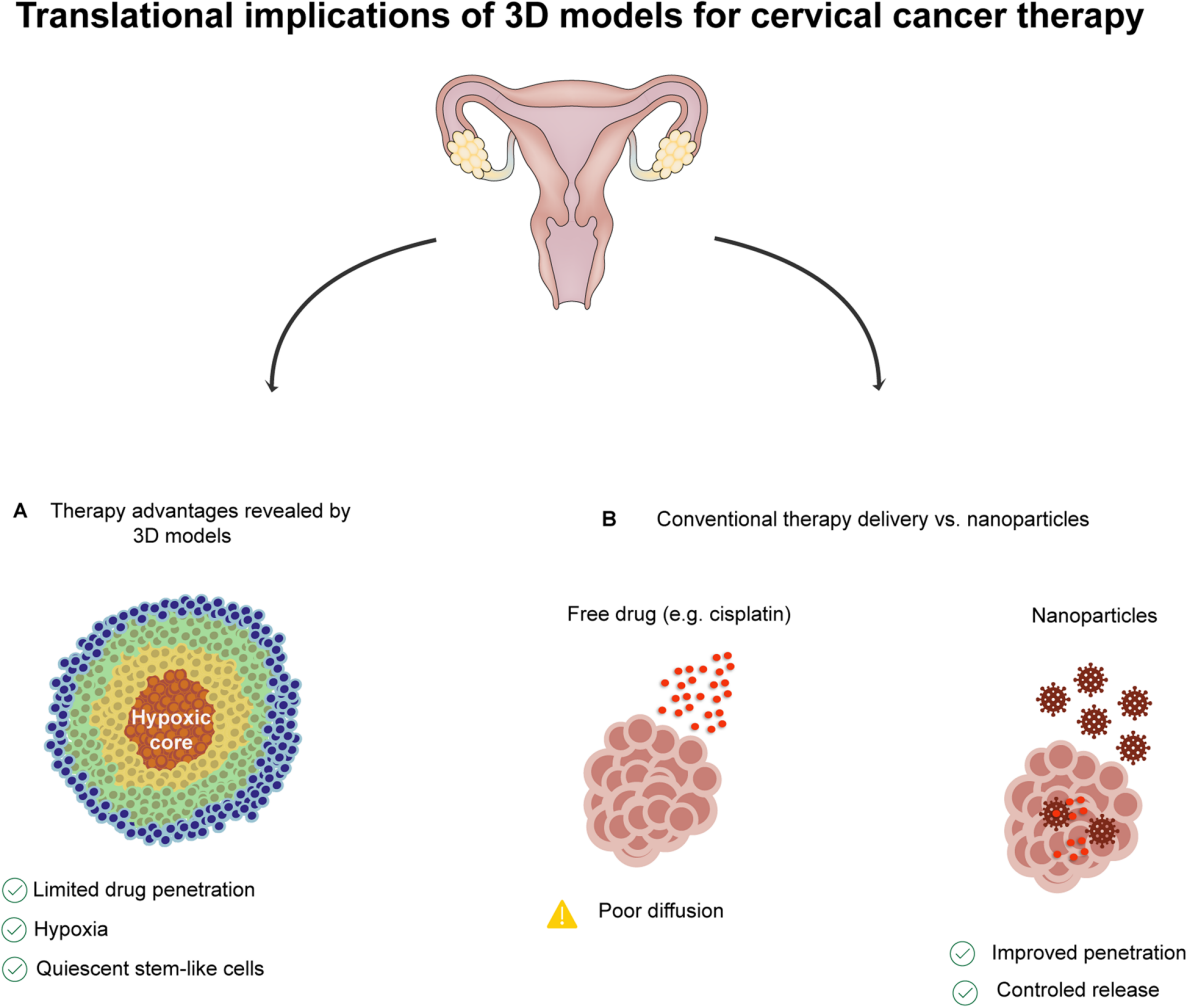
Translational implications of three-dimensional (3D) models for therapeutic response in cervical cancer. **A** Three-dimensional spheroids and organoids recapitulate key microenvironmental barriers to treatment efficacy, including limited drug penetration, hypoxic cores, and the persistence of quiescent, stem-like cell populations that are intrinsically less sensitive to cytotoxic therapies. These features are largely absent in conventional two-dimensional cultures. **B** Comparison of conventional chemotherapy delivery using freely diffusible agents (e.g., cisplatin) versus nanoparticle-based formulations. Free drugs exhibit limited penetration and preferential accumulation in peripheral tumor regions, whereas nanoparticles achieve improved intratumoral penetration and controlled release, enhancing drug exposure within hypoxic and therapy-resistant niches

Nanoparticle uptake mechanisms also differ markedly between 2D and 3D systems. In 2D cultures, uptake is relatively uniform and dominated by clathrin-mediated endocytosis, with high per-cell nanoparticle accumulation due to direct exposure. In contrast, 3D spheroids exhibit pronounced spatial heterogeneity: proliferative cells at the periphery display higher receptor expression and uptake rates, whereas hypoxic or quiescent cells in inner regions show reduced internalization and significantly lower intracellular nanoparticle levels [101, 107]. Quantitative analyses consistently report reduced average uptake per cell and increased effective IC₅₀ values in 3D systems compared with matched 2D cultures, closely mirroring *in vivo*behavior. Microenvironmental stress further reshapes endocytic pathways. Hypoxia and metabolic reprogramming alter membrane composition and endocytic machinery, potentially reducing constitutive uptake routes while biasing cells toward macropinocytosis or alternative uptake mechanisms. In HPV-positive cervical cancer models, E6/E7-driven metabolic alterations combined with hypoxia have been shown to modulate uptake behavior in CSC-enriched spheroids, emphasizing the importance of considering viral oncogene context when designing nanoparticle delivery strategies [101, 104]. Recent methodological advances—including spatially resolved imaging, flow cytometric analysis of dissociated spheroid layers, and computational tools for spatiotemporal uptake quantification—now enable rigorous comparison of nanoparticle behavior across 2D and 3D systems. Together with emerging cervical organoid models derived from the transformation zone that preserve HPV-associated architecture and molecular features, these platforms provide a more realistic framework for evaluating nanoparticle-based therapies targeting CSCs and overcoming therapeutic resistance (Fig. 3).

## Conclusions and future perspectives

Cervical cancer exemplifies how viral oncogenesis, epithelial plasticity, and microenvironmental regulation converge to drive therapeutic resistance and disease recurrence. Although persistent infection with high-risk human papillomavirus (HPV) is a necessary initiating event, mounting evidence indicates that treatment failure cannot be explained by viral genotype, histology, or clinical stage alone. Instead, resistance emerges from a dynamic interplay between stem-like cell states, reversible drug tolerance, metabolic adaptation, and tumor microenvironmental cues—processes that are fundamentally spatial and temporal in nature. Three-dimensional (3D) culture systems have been instrumental in reshaping this conceptual framework. By preserving tissue architecture, lineage hierarchies, and microenvironmental gradients, spheroids, organoids, and matrix-informed platforms reveal adaptive cell states that are largely masked in two-dimensional models. These systems demonstrate that therapy resistance in cervical cancer is frequently driven by non-genetic, reversible programs, including quiescence, redox adaptation, and stem-like plasticity, which enable tumor cells to survive chemoradiotherapy and reinitiate growth following treatment withdrawal. Importantly, 3D models also preserve HPV episomes and sustain viral oncogene expression, allowing direct interrogation of viral–host interactions under physiologically relevant conditions. From a translational perspective, advanced 3D models are redefining preclinical evaluation of therapeutic strategies. Organoid-based platforms have shown superior predictive power for drug response compared with conventional monolayer cultures and provide a robust framework for identifying context-dependent vulnerabilities. This is particularly relevant for emerging approaches such as nanoparticle-based delivery systems, whose penetration, uptake, and efficacy are strongly influenced by extracellular matrix composition, hypoxia, and cellular heterogeneity—features that are faithfully reproduced only in 3D settings. Integrating stem-cell–enriched organoids with biomaterial-informed matrices and microfluidic technologies further expands the capacity to model treatment-induced stress responses and resistance evolution in real time.

Despite these advances, several challenges remain. Standardization of organoid derivation protocols, maintenance of genetic and phenotypic stability during long-term culture, and incorporation of stromal, immune, and vascular components are critical next steps. In addition, systematic integration of spatially resolved omics, functional lineage tracing, and quantitative modeling will be required to disentangle transient adaptive states from stable resistance programs. Evidence supports a complementary rather than hierarchical relationship between experimental systems. Three-dimensional cultures provide spatial and cellular organization, organ-on-chip platforms enable dynamic perfusion and multicellular interactions, and *in vivo*models capture systemic physiology [94, 95]. Integrative approaches combining these platforms are therefore essential to reproduce tumor complexity and improve translational predictability. Recent work has emphasized how immune–tumor interactions, microenvironmental signaling, and treatment response are best understood through multi-platform modeling strategies Addressing these limitations will be essential for translating mechanistic insights from 3D models into clinically actionable strategies.

Looking forward, the convergence of HPV-informed biology, advanced 3D culture systems, and precision therapeutic platforms offers a powerful opportunity to shift cervical cancer research from descriptive marker-based models toward dynamic, function-driven frameworks. By capturing the reversible and spatially organized nature of therapy resistance, these approaches hold promise not only for improving treatment stratification and drug development but also for identifying intervention windows that prevent recurrence and ultimately improve long-term outcomes for patients with cervical cancer.

## Data Availability

Data sharing is not applicable to this article as no new data were generated or analyzed in this study.

## Abbreviations

SCC: Squamous Cell Carcinoma
TZ: Transformation Zone
HPV: Human Papillomavirus
CICs: Cancer-Initiating Cells
CSCs: Cancer Stem-like Cells
EMT: Epithelial-Mesenchymal Transition
TME: Tumor Microenvironment
ECM: Extracellular Matrix
3D: Three-Dimensional
2D: Two-Dimensional
PDOs: Patient-Derived Organoids
AI: Artificial Intelligence
ABC: ATP-Binding Cassette (transporters)
ALDH1: Aldehyde Dehydrogenase 1
GSH: Glutathione
HR: Homologous Recombination
NHEJ: Non-Homologous End Joining
OXPHOS: Oxidative Phosphorylation
ROS: Reactive Oxygen Species
CAR-T: Chimeric Antigen Receptor T-cells
MDSCs: Myeloid-Derived Suppressor Cells
PD-L1: Programmed Death-Ligand 1
TAMs: Tumor-Associated Macrophages
TCR: T-cell Receptor
TLR: Toll-like Receptor
Tregs: Regulatory T cells
